# *Melaleuca styphelioides* Sm. Polyphenols Modulate Interferon Gamma/Histamine-Induced Inflammation in Human NCTC 2544 Keratinocytes

**DOI:** 10.3390/molecules23102526

**Published:** 2018-10-02

**Authors:** Ferdaous Albouchi, Rosanna Avola, Gianluigi Maria Lo Dico, Vittorio Calabrese, Adriana Carol Eleonora Graziano, Manef Abderrabba, Venera Cardile

**Affiliations:** 1 Laboratoire Matériaux-Molécules et Applications, University of Carthage, IPEST, B.P. 51 2070, La Marsa, Tunisia; ferdaous.albouchi@gmail.com (F.A.); abderrabbamanef@gmail.com (M.A.); 2 Faculte des Sciences de Bizerte, University of Carthage, Jarzouna, 7021, Bizerte, Tunisia; 3 Department of Biomedical and Biotechnological Sciences, Section of Physiology, University of Catania, Via Santa Sofia, 97-95123 Catania, Italy; rosanna.avola@unict.it (R.A.); acegraz@unict.it (A.C.E.G.); 4 Istituto Zooprofilattico Sperimentale della Sicilia "A. Mirri", Via Gino Marinuzzi 3, 90129 Palermo, Italy; gigilodico@gmail.com; 5 Department of Biomed & Biotech Sciences, School of Medicine, University of Catania, Via Santa Sofia 97, 95125 Catania, Italy; calabres@unict.it

**Keywords:** *Melaleuca styphelioides*, polyphenols, LC/MS-MS, anti-oxidant activity, anti-inflammatory activity, keratinocytes

## Abstract

*Melaleuca styphelioides*, known as the prickly-leaf tea tree, contains a variety of bioactive compounds. The purposes of this study were to characterize the polyphenols extracted from *Melaleuca styphelioides* leaves and assess their potential antioxidant and anti-inflammatory effects. The polyphenol extracts were prepared by maceration with solvents of increasing polarity. The LC/MS-MS technique was used to identify and quantify the phenolic compounds. An assessment of the radical scavenging activity of all extracts was performed using 2,2-diphenyl-1-picrylhydrazyl (DPPH), 2,2′-azinobis-(3-ethylbenzothiazoline-6-sulphonate) (ABTS^+^), and ferric reducing antioxidant power (FRAP) assays. The anti-inflammatory activity was determined on interferon gamma (IFN-γ)/histamine (H)-stimulated human NCTC 2544 keratinocytes by Western blot and RT-PCR. Compared to other solvents, methanolic extract presented the highest level of phenolic contents. The most frequent phenolic compounds were quercetin, followed by gallic acid and ellagic acid. DPPH, ABTS^+^, and FRAP assays showed that methanolic extract exhibits strong concentration-dependent antioxidant activity. IFN-γ/H treatment of human NCTC 2544 keratinocytes induced the secretion of high levels of the pro-inflammatory mediator inter-cellular adhesion molecule-1 (ICAM-1), nitric oxide synthase (iNOS), cyclooxygenase-2 (COX-2), and nuclear factor kappa B (NF-κB), which were inhibited by extract. In conclusion, the extract of *Melaleuca styphelioides* leaves is rich in flavonoids, and presents antioxidant and anti-inflammatory proprieties. It can be proposed as a useful compound to treat inflammatory skin diseases.

## 1. Introduction

Inflammation is a complicated series of protective responses, involving several cell types and various putative modulators and mediators [1]. Skin provides both chemical and physical barriers and contains the cellular components of a rapid innate immune response [2], which protects the organism against external invasions.

Keratinocytes are the major constituents of the epidermis and appear to be exposed to stressful conditions such as toxins and microbes. They are also the major players of the complex response in the skin, conducting the activation of a diversity of Toll-like receptors [3,4]. Histamine (H) and interferon gamma (IFN-γ) can induce inflammatory responses in keratinocytes, highlighted by the activation of the pro-inflammatory mediators, such as the nuclear factor kappa B (NF-κB), inter-cellular adhesion molecule-1 (ICAM-1), nitric oxide synthase (iNOS), and cyclooxygenase-2 (COX-2) [5,6,7]. Inhibitors of these mediators are extensively used to treat several inflammatory diseases. However, the majority of such anti-inflammatory drugs (steroidal or non-steroidal molecules) are highly toxic, and their use is often associated with harmful effects on the gastrointestinal tract, such as mucosal lesions, bleeding, and peptic ulcers [8].

Currently, a regular growing interest in plant polyphenols is proposed as an alternative to treat skin inflammatory diseases. Plant polyphenols are one of the greatest groups, with the largest chemical diversity. In addition, polyphenols are widely used in traditional medicine to treat several skin diseases, like vitiligo, psoriasis [9], and atopic dermatitis [10], as well as to accelerate skin wound healing [9]. However, the mechanisms by which plant phenolic compounds exert their anti-inflammatory effect are poorly understood. The potential anti-inflammatory effects of polyphenols have been attributed to either their free radical scavenging activities or classical chain-breaking, antioxidant, and transition metal chelating activity [11]. Other in-vitro studies using skin cells suggest that polyphenols inhibit the activation of cellular functions by multiple mechanisms, such as the modulation of intracellular signal transduction and transcription of a number of genes, direct interaction with several receptors, and post-translational modulation of enzymatic activities [12,13]. It is well-documented that polyphenols inhibit major pro-inflammatory skin enzymes, such as COX, LOX, iNOS, NADPH oxidase, NF-κB, ICAM-1, and PLA2 [12]. The anti-inflammatory mechanism of plant polyphenols depends on various factors, such as chemical structure, synergy of other phenols, cell types, and inductor used [12].

*Melaleuca (M.) styphelioides* Sm. belongs to the *Melaleuca* genus, commonly known as the prickly-leaf tea tree. The *Melaleuca* genus, or tea tree, is presented by approximately 260 described species native to Australia and is widespread in Southeast Asia, the Caribbean, and the Southern United States. In different parts of world, tea tree is used in traditional medicine as a treatment for insect bites, bruises, skin infections, flu and colds, acne vulgaris, psoriasis, inflammation, dermatitis, an antimicrobial agent, and as an insect repellant. Moreover, *Melaleuca* species are used in the manufacture of cosmetics, such as shampoos, soaps, and some cosmetic products [14]. *M. styphelioides* is well-known species that has been reported to have the strongest production of scented essential oil and tannins, as well as amount of flavonoid compounds and phenolic acids [15]. Several studies have reported the antioxidant, antimicrobial, hepatoprotective, and anti-proliferative activities of the essential oil and extract isolated from *M. styphelioides* leaves [15,16,17,18].

Here, we aim to (i) characterize and quantify the polyphenol compounds present in *M. styphelioides* leaves, and (ii) evaluate their antioxidant and anti-inflammatory activities in IFN-γ/H-induced inflammation of human NCTC 2544 keratinocytes. To our knowledge, this is the first report that demonstrates the antioxidant and anti-inflammatory activities of polyphenols extracted by *M. styphelioides* leaves.

## 2. Results and Discussion 

In this work, four solvents of increasing polarities were chosen for the evaluation of phenolic compounds contents of *M. styphelioides* leaves, namely hexane, diethyl ether, ethyl acetate, and methanol.

The yields of total phenol, flavonoids, and tannins from *M. styphelioides* leaf extracts are shown in Table 1. The maximum content of phenols total (PT) (142.7 ± 3.15 mg gallic acid equivalents (GAE)/g dry extract) and flavonoid total (FT) (31.54 ± 1.99 mg quercetin equivalents (QE)/g dry extract) was obtained in the MeOH extract. Lower amounts were found in the ethyl acetate and Et_2_O extracts, and even lower amounts in the hexane extract. The highest amount of TC (19.9 ± 2.9 mg Eq Catéchine/g dry extract) was determined in the EtOAc and MeOH extracts (15.2 ± 1.9 mg Eq Catéchine/g dry extract), while no TC was present in Et_2_O and hexane extracts (Table 1). The important phenolic compounds extraction yields, found in the MeOH extract, has been attributed to its high solubility, low toxicity, medium polarity, and high extraction capacity. Our results are in agreement with the previously report of Al-Sayed et al. [15], where the authors report a higher amount of phenolic and flavonoid components in the MeOH extract of *M. styphelioides* leaves.

**Figure 1:**
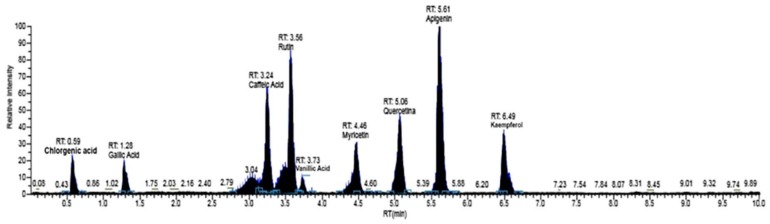
Representative LC/MS-MS of phenolic components in leaf methanolic extract of *M. styphelioides* leaves.

Based on the optimization condition of LC/MS-MS, the MeOH extract of *M. styphelioides* leaves was subjected to identification and quantification of the polyphenol components, in order to better discuss its biological potential. The detailed phenolic composition was determined using our standard library information (peak retention time, [M − H^−^] (m^2^), and LC-MS/MS data). *M. styphelioides* leaf extract showed the presence of several types of phenols belonging to diverse phenolic families, such as phenolic acids and flavonoids. Fifteen phenolic compounds were identified and quantified, with a range of 3.04–5.61 min retention times (RT) in negative polarity mode (Figure 1 and Table 2). Qualitatively, the phenolic profile included seven phenolic acids (vanillic acid, gallic acid, caffeic acid, syringic acid, chlorogenic acid, ferulic acid, and ellagic acid), nine flavonoids (apigenin, kaempferol, myricetin, naringenin, quercetin, luteolin, pinocembrin, hesperidin, and rutin). In quantitative terms, *M. styphelioides* polyphenol extract contained a rich source of bioactive phenols, mainly phenolic acids (25%) and flavonoids (70.6%). Most of the detected flavonoids corresponded to quercetin (53.99%) and apigenin (4%), followed by kaempferol (3.2%). The major phenolic acid compound found in metanolic extract was gallic acid (13.5%), followed by ellagic acid (6.3%) and vanillic acid (4.3%). 

Previous studies regarding the phenolic components of *Melaleuca* plants have reported a rich composition of bioactive compounds, such as ellagitannins and flavonoids [15,16]. According to Al-Sayed et al. [15,16], gallic acid, kaempferol-3-*O*-α-l-rhamnopyranoside, pedunculagin, pterocarinin A, tellimagrandin I, casuarinin, cellimagrandin II, 1,2,3,6-tetra-*O*-galloyl-β-d-glucopyranose and 1,2,3,4,6-penta-*O*-galloyl-β-d-glucopyranose were the representative polyphenol compounds in *M. styphelioides* leaves. Al-Abd et al. [19] studied the phenolic composition of *Melaleuca cajuputi* leaves and indicate the presence of gingerol, caffeic acid, phenyl ester, aspidin, trans-2,3,4-trimethoxycinnamate, methyl orsellinic acid ester, ethyl ester, 5,6,3′-trimethoxyflavone, epigallocatechin 3-*O*-(4-hydroxybenzoate), polygonolide, and metyrosine. Compared to the aforementioned studies, our results show striking differences, presumably due to the species, origin, and analytical conditions [19]. Nevertheless, knowledge of *M. styphelioides* polyphenol compounds was extended by the identification of fourteen compounds (vanillic acid, gallic acid, caffeic acid, syringic acid, chlorogenic acid, ferulic acid, ellagic acid, apigenin, kaempferol, myricetin, naringenin, quercetin, luteolin, pinocembrin, hesperidin, and rutin) not previously reported in the *M. styphelioides* plant. From this finding, *M. styphelioides* appears as a source of bioactive compounds, justifying its pharmacological properties. Almost all of the identified phenolic compounds were well-known as antioxidants and anti-inflammatories.

The ability of *M. styphelioides* polyphenol extract to reduce the DPPH, ABTS radicals, and ferric reducing antioxidant power (FRAP) are shown in Table 3. DPPH and ABTS assays are frequently used as inexpensive, valid, and easy assays to evaluate the radical scavenging capacity of antioxidants. All extracts, excluding hexane extract, showed a concentration-dependent scavenging activity. The methanolic extract proved to be the highest DPPH and ABTS scavenger, with mean EC_50_ values of 22.13 and 21.39 µg/ml, respectively, followed by diethyl ether extract (73.24 ± 2.811 and 52.22 ± 1.40, respectively) and ethyl acetate (119.15 ± 1.66 and 75.84 ± 1.22, respectively). These results reflect the hydrogen-donating ability of the methanolic extracts from *M. styphelioides* leaves. On the other hand, the higher DPPH and ABTS scavenging activities of methanolic samples are most likely attributed to their higher total phenolic contents.

The ferric reducing power method involves the reduction of the Fe(III)-tripyridyltriazine (Fe(III)-TPTZ) complex to Fe(II)-tripyridyltriazine (Fe(II)-TPTZ) at a low pH by electron-donating antioxidants, resulting in the absorbance increase at λ = 593 nm. As presented in Table 3, the FRAP values of the methanolic extract (3.66 mM FeSO_4_/g dw) was significantly (*p* < 0.05) higher than that ethyl acetate extract (0.85 mM FeSO_4_/g dw). The non-polar extracts with diethyl ether and hexane did not present any reducing power activity. As displayed in Table 3, it is clearly observed that the methanolic extracts can be considered as effective scavengers of DPPH and ABTS radicals, as well as potent reducing agents.

From a mechanistic standpoint, the observed antioxidant activity reflects the ability of the test extracts to donate electrons or hydrogen atoms to inactivate radical species [20]. Such properties have been reported for numerous phenolic compounds, namely gallic acid, syringic acid, chlorogenic acid, caffeic acid, rutin, chlorogenic acid, luteolin glucoside, apigenin derivative, and quercetin [21,22]. In addition, our results confirm the potential therapeutic uses of *M. styphelioides* extract for inflammatory diseases and cancers [15,16]. Moreover, our data, supported by analytical LC/MS-MS, are in agreement with those of Al-Sayed et al. [15,16], although their data are limited to evaluation of the antiradical activity of methanolic extracts from *M. styphelioides* leaves by DPPH assay only.

Keratinocytes are the major players of the innate immune response in the skin. They promote inflammation via expression of several key bio-mediators. However, these cells can also adapt an anti-inflammatory behavior, in order to stop acute inflammation and retrieve a steady state through the secretion of immuno-modulating agents, such as ICAM-1, COX-2, iNOS, and NF-κB. Human keratinocytes have been reported to be useful cellular barrier models for both host–pathogen interaction studies and synthetic or natural compound anti-inflammatory screening. Here, we used NCTC 2544 cells to assess the effect of *M. styphelioides* phenolic extract on IFN-γ/H-induced inflammation.

The cell toxicity of polyphenol extracts was assessed by an MTT assay after 72 h treatment with different concentrations of *M. styphelioides* polyphenols. The MTT assay revealed that the MeOH extract had a lower toxicity compared to other extracts (data not shown). Moreover, the dose of 50 µg/mL of M. MeOH extract showed no toxicity and a high antioxidant property (data not shown). Thus, we chose to use 50 µg/mL concentration in NCTC 2544 cells treated with IFN-γ/H, in order to investigate the potential anti-inflammatory activity of the M. MeOH extract.

**Figure 2:**
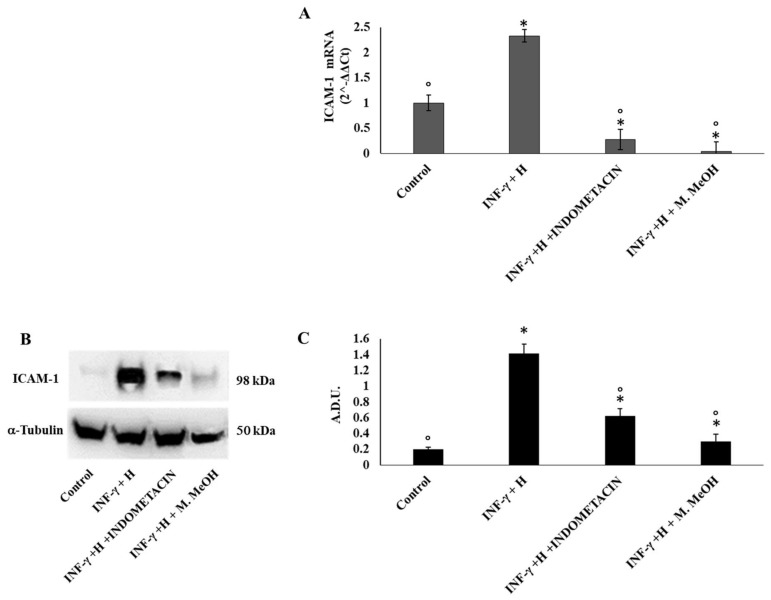
Inter-cellular adhesion molecule-1 (ICAM-1) mRNA expression (**A**) and protein production (**B**, and **C**). ICAM-1 mRNA expression was determined by RT-PCR (**A**), and ICAM-1 protein production was determined using Western blot (**B**: representative immunoblot; **C:** protein expression calculated as Arbitrary Densitometric Units; A.D.U.) in NCTC 2544 72 h after the addition of M. MeOH (50 µg/mL) with INF-γ + H. * Significantly different than control; ° significantly different from INF-γ/H-treated samples (*p* < 0.05).

In order to examine the anti-inflammatory activity of M. MeOH extract in human keratinocytes, we measured in IFN-γ/H-treated NCTC 2544 cell proteins and mRNA gene expression levels of ICAM-1 and COX-2 by Western blot and RT-PCR, respectively. As shown in Figure 2A, IFN-γ/H treatment for 72 h significantly increased the level of mRNA expression of ICAM-1, whereas M. MeOH extract treatment (50 µg/mL) for 72 h inhibited the IFN-γ/H induced expression of ICAM-1. Compared to M. MeOH, indomethacin (10 µM) inhibited the mRNA expression of ICAM-1 to a higher degree. The Western blot confirmed the results obtained by RT-PCR (Figure 2B). In inflamed keratinocytes, the expression of ICAM-1 is always increased providing retention and activation of lymphocytes (CD4^+^ and CD8^+^) in the epidermis. Moreover, the regulation of ICAM-1 expression on keratinocytes using phenols is considered as a recent strategy for the treatment of skin inflammatory diseases [23].

**Figure 3:**
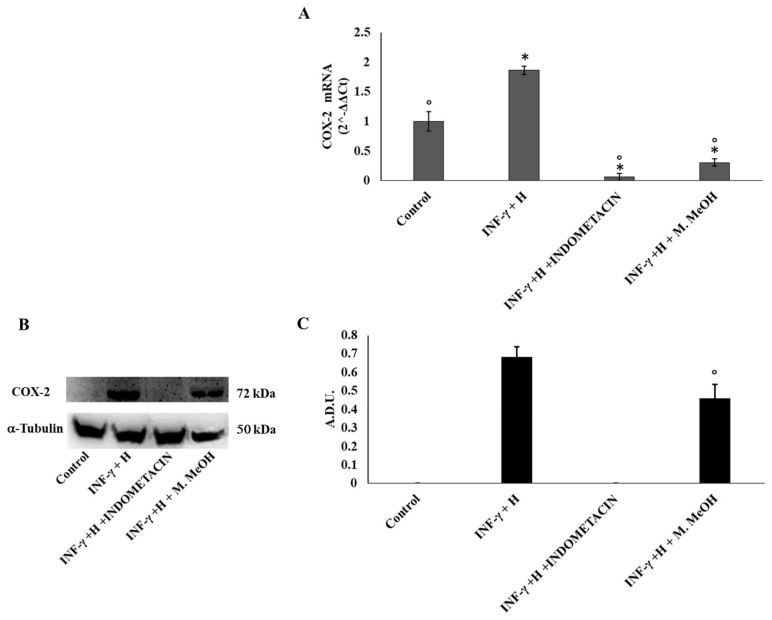
Cyclooxygenase-2 (COX-2) mRNA expression (**A**) and protein production (**B,** and **C**). COX-2 mRNA expression was determined by RT-PCR (**A**), and COX-2 protein production was determined using Western blot (**B**: representative immunoblot; **C:** protein expression calculated as Arbitrary Densitometric Units; A.D.U.) in the NCTC 2544 72 h after the addition of M. MeOH (50 µg/mL) with INF-γ + H. * Significantly different than control; ° significantly different than INF-γ/H-treated samples (*p* < 0.05).

Prostaglandins are also one of the major classes of mediators in the inflammatory response. They are generated from arachidonate by the action of cyclooxygenase isoenzymes (COX-1 and COX-2) [24]. In most tissues, COX-1 is constitutively expressed, whereas COX-2 is highly inducible by a variety of inflammatory and tumor-promoting stimuli, and is constitutively upregulated in skin carcinomas [25]. Our data demonstrates that COX-2 mRNA expressions were reduced significantly by the M. MeOH extract treatment in IFN-γ/H-induced inflammation, whereas its effect on protein levels was moderate compared to indomethacin (Figure 3).

**Figure 4:**
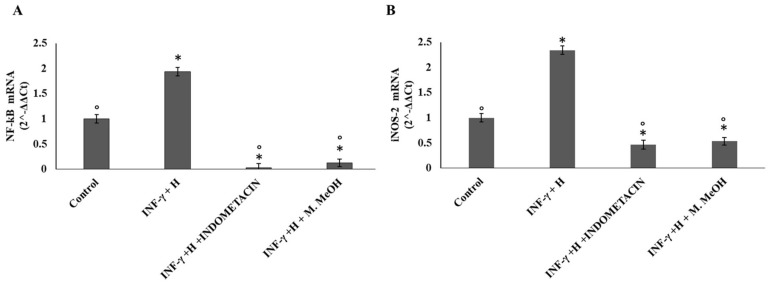
Nuclear factor kappa B (NF-κB) (**A**) and nitric oxide synthase (iNOS) mRNA expression (**B**). The mRNA expression was determined by RT-PCR in the NCTC 2544 for 72 h after the addition of M. MeOH (50µg/mL) with INF-γ/H. * Significantly different than control; ° significantly different from INF-γ/H-treated samples (*p* < 0.05).

The expression of the inducible nitric oxide synthase (iNOS) is one of the direct consequences of an inflammatory process. Several methods such as RT-PCR, immunocytochemistry, and Western blot, have been used to describe the iNOS expression in many chronic human inflammatory diseases. The iNOS mRNA level is mainly regulated at the transcriptional level but also by other post-transcriptional regulatory mechanisms. Phenolic acid and flavonoids have been reported to inhibit iNOS expression in several cell types, including endothelial cells, epithelial cells, and macrophages, yet in some cell types like chondrocytes [26]. Here, our data showed that the iNOS mRNA level was increased after cell stimulation with IFN-γ/H for 72 h, with respect to the non-treated control. A significant inhibition of iNOS mRNA expression was observed when the NCTC 2544 cells were co-treated with IFN-γ/H and 50 μg/mL of M. MeOH extract. Compared to M. MeOH extract, indomethacin weakly reduced iNOS mRNA levels (Figure 4B).

In addition, we examined the influence of M. MeOH extract treatment on IFN-γ/H-induced NF-κB mRNA expression. As shown in Figure 4A, NF-κB mRNA expression was increased after stimulation with IFN-γ and H for 72 h. Significant inhibition of NF-κB mRNA expression was observed when the NCTC 2544 was co-treated with IFN-γ/H and 50 μg/mL of M. MeOH extract. Compared to M. MeOH extract, indomethacin weakly reduced the NF-κB mRNA levels. Several studies have provided that the keratinocytes, when exposed to IFN-γ and histamine, activate NF-κB, leading to the expression of inflammatory genes. More than 150 genes have been identified that are regulated by NF-κB activation. These genes include iNOS, ICAM-1, COX-2, and chemokines [27]. Therefore, NF-κB is primarily an inducer of inflammatory cytokines, and its inhibitors could be useful as anti-inflammatory agents. In the present study, our data showed that M. MeOH extracts inhibited the mRNA expression of NF-κB in IFN-γ/H-activated NCTC 2544 keratinocytes. In addition, iNOS, ICAM-1, and COX-2 mRNA expression were markedly decreased with the M. MeOH extract treatment. Compared with indomethacin, the effect of M. MeOH extract was either higher or similar, probably due to the different active compounds present in *M. styphelioides* extracts.

In summary, our work represents the first report of an anti-inflammatory effect of *M. styphelioides* extract in IFN-γ/H-stimulated human NCTC 2544 cells. M. MeOH extract inhibited the expression of pro-inflammatory mediators by inflamed NCTC 2544 cells. This inhibition was primarily mediated by the modulation of the major cellular effectors of inflammation, such as NF-κB, iNOS, iCAM-1, and COX-2.

Quercetin, the major flavonoid molecule found in M. MeOH extract (53.99%), has been reported to down-regulate the activation of NF-κB, ICAM-1 [23], iNOS [28], and COX-2 [29]. Gallic acid (13.5%) and ellagic acid (6.3%), two important compounds of M. MeOH extract, have been reported to produce a beneficial antioxidant effect by regulating several pathways. These include inhibition of COX-2 [30] and cytokines activated by the NF-κB pathway [31]. All compounds identified and quantified in M. MeOH extract, like vanillic acid [32,33], apigenin [34], syringic acid [35], kaempferol [36], and rutin [37] have also been shown to have a potential anti-inflammatory activity. Our data suggest that each molecule may have contributed to the anti-inflammatory effect of the M. MeOH extract. However, additional work is needed to better characterize the contribution of each compound, even though the phenolic compounds probably act synergistically to generate a more potent anti-inflammatory effect. 

## 3. Conclusions

Previously, *M. styphelioides* methanolic extract has been investigated for anti-proliferative and anti-cancer effects [38], but to our knowledge, we have shown for the first time that *M. styphelioides* extract also has an anti-inflammatory property. The cytoprotective, antioxidant, and anti-inflammatory potential of *M. styphelioides* can be of great interest as an effective alternative for the treatment and prevention of inflammatory diseases.

## 4. Materials and Methods

### 4.1. Chemicals and Reagents

All chemicals and reagents were either analytical-reagent or HPLC grade. Ultrapure deionized water, with a resistivity of 18.2 MΩ cm, was obtained from a Milli-Q^®^ Integral water purification system with a Q-pod purchased from Millipore (Bedford, MA, USA). Acetic acid, acetonitrile, and 2-propanol were purchased from VWR International S.r.l. (Milan, Italy); hydrochloric acid and sodium hydroxide were purchased from Carlo Erba (Milan, Italy). The methanol HPLC gradient grade was obtained from Merck (Darmstadt, Germany). Standard solutions of vanillic acid, trans-ferulic acid, syringic acid, myricetin, naringenin, pinocembrin, luteolin, and hesperidin were purchased from Extrasynthese (Genay Cedex, France). Chlorogenic acid was purchased from HWI Analytik GmbH (Rülzheim, Germany). Gallic acid, caffeic acid, apigenin, quercetin, kaempferol, catechin, epicatechin, indometacine, histamine, and anti-α-tubulin antibody were purchased from Sigma-Aldrich S.r.l. (Milan, Italy). The IFN-γ was obtained from Pepro Tech EC (London, England). The anti-ICAM-1 antibody (sc-51632) was purchased from Santa Cruz Biotechnology (Milan, Italy), anti-COX-2 (35-8200) from Thermo Fisher Scientific (Milan, Italy), and -α- tubulin (T6074) from Sigma-Aldrich (Milan, Italy).

### 4.2. Plant Material and Extracts Preparation

Leaves of *M. styphelioides* were collected from healthy trees in April 2016 from the botanical garden of the National Institute of Agricultural Research (INRAT, Tunis), Tunisia. Specimens were deposited at the Herbarium of the Department of Botany in the cited institute. Leaves were air-dried at room temperature (20 ± 2 °C) for one week, ground using a Retsch blender mill (Normandie-Labo, Normandy, France), sifted through a 0.5 mm mesh screen to obtain a uniform particle size, and subsequently assessed for their phenolic composition. Dried and ground leaves *M. styphelioides* were defatted three times by maceration with n-hexane for 48 h. The defatting process was used to extract lipophilic pigments and oil from lipid-containing samples and facilitate the polyphenol extract. The defatted samples were extracted with solvents of increasing polarity (diethyl ether, ethyl acetate, and methanol), sonicated for 30 min, and macerated for 24 h. The macerated organic extracts were filtered through Wattman filter paper, centrifuged for 10 min at 3000× *g*, and concentrated under reduced pressure in a Heidolph rotary evaporator. The obtained extracts were kept and stored at 4 °C until further analysis.

### 4.3. Phytochemical Analysis

#### 4.3.1. The Phytochemical Screening

The phytochemical screening of total phenol, flavonoids, and tannins was determined according to the procedure reported by El Euch et al. [39].

#### 4.3.2. LC/MS-MS Analysis

The mixture of polyphenols was determined according to previous work [40], using a Transcen II System with Multi-channel and Turbo Flow Technology (Dionex–Thermo Fisher Scientific) connected to a Q-Exactive Plus Hybrid Quadrupole-Orbitrap Mass Spectrometer (Thermo Fisher Scientific) equipped with HESI (heated electro spray ionization), used in negative polarity modes. The samples were extracted and purified using a Cyclone P column (50 mm × 0.5 m, 60 µm particle size, 60 Å pore size; Thermo Fisher Scientific). A Hypersil Gold (2.1 mm × 100 mm, 1.7 µm particle size) column was employed as the analytical separation column. The mobile phase consisted of eluent A (30 mM ammonium acetate (pH 5)), eluent B (methanol), eluent C (water containing 0.5% formic acid), and eluent D (acetonitrile/acetone/2 propanol (4:3:3)). Mobile phases A and B were used to optimize the chromatographic resolution. Mobile phases B, C, and D were required for purification in turbo flow. Extracts and standards were dissolved in the mobile phase A (ratio 1:10). The injection volume was 5 µL, and elution was performed at the rate of 0.2 mL/min with a gradient program as follows: 0–2 min with 95% A and 5% B; 4 min with 60% A and 40% B; 6 min with 0% A and 100% B; 9 min with 0% A and 100% B; 11.5 min with 95% A and 5% B; 14 min with 95% A and 5% B; and 18 min with 95% A and 5% B. The acquisition time was 10 min. Eluted components were detected by MS, used in negative polarity modes, using the ion source parameters as follows: sheath gas flow rate of 35 (arbitrary units); aux gas flow rate of 10 (arbitrary units); spray voltage at 3.50 kV; capillary temperature at 300 °C; tube lens voltage of 55 V; heater temperature of 305 °C; scan mode at full scan; scan range (*m*/*z*) at 100–700; microscans at 1 *m*/*z*; a positive resolution of 70,000; an FT automatic gain control (AGC) target of 3 × 10^6^; a maximum IT of 100 ms; a negative resolution of 35,000; an automatic gain control (AGC) target of 1 × 10^6^; and a maximum IT of 100 ms. The chromatographic parameters were as follows: column temperature at 30 °C and sample temperature at 6 °C. The auto-sampler sample holder temperature was maintained at 7 °C. The data analyses were performed using a Thermo Scientific XCalibur (Thermo Fisher Scientific) version 4.0 software and Qual and Quant Browser, and the concentration of the compounds were calculated using calibration curves; the results are expressed as calibration curves and as µg/kg of dry weight (DW). This method was validated according to the norm EN ISO/IEC 17025:2005. The limits of detection and quantification (LoDs and LoQs) were determined by the 3σ and 10σ approach [41,42]. The calibration curve was constructed with six standard additions (0.05, 0.1, 0.2, 0.5, 1, and 2 mg/L), and was checked using the r^2^ value. The linearity range was considered to be acceptable when r^2^ was greater than 0.99 in the peak areas versus the concentration. A pool of 15 blank samples spiked with final concentrations of 5 µg/kg for all elements were analysed. The results were between 10 µg/kg for the LOD and 20 µg/kg for the LOQ for single analytes. The limit of repeatability and recovery has been evaluated, with the spike samples at three different concentration levels (0.1, 0.5, and 1 mg/L). The results were satisfactory for the limit of repeatability (metrological approach), less than the double value of the expanded uncertainty. The recovery was between 71% and 119%. The validation allowed us to identify the uncertainty contributions in order to calculate the expanded uncertainty [43]. The results show that the expanded uncertainty is less than 22% in all the analyzed levels.

### 4.4. Anti-Oxidant Activity Determination

The DPPH and ABTS radical scavenging activity was assessed by the method described by El Euch et al. [39]. The antioxidant effect was expressed as IC_50_, which is the amount of extract required to scavenge the initial DPPH or ABTS radical by 50%, and is expressed as per mg of the sample [39]. The reducing power (FRAP) was determined by the method described in [44]. The results were expressed as FeSO_4_·7H_2_O-equivalent mM per gram of dry extract (mM/g DE), using a calibration curve.

### 4.5. Anti-Inflammatory Activity Evaluation

#### 4.5.1. Cell Culture and Treatment

The NCTC 2544 keratinocyte cell line was obtained from Interlab Cell Line Collection (Genoa, Italy). Cells were grown in Minimum Essential Medium (Sigma-Aldrich, Milan, Italy), containing 10% foetal bovine serum (FBS), 100 μg/mL streptomycin, and 100 U/mL penicillin. The cells were then incubated at 37 °C in a humidified, 95% air 5% CO_2_ atmosphere. The culture medium was changed every 2–3 days. For experiments, cells were trypsinized, counted, and plated in six- or 96-well plates. Cells were either treated or not treated with 200 U mL^−1^ of IFN-γ and 10^−4^ M of H, in the presence or absence of different concentrations of *M. styphelioides* polyphenol extracts (5, 10, 25, 50, and 75 μg/mL). Commercially available indomethacin was used as a positive control at 10 µM. After 72 h, each sample was tested for the experiments described below.

#### 4.5.2. Cell Viability

The phenol extracts’ cytotoxicity was determined by an MTT assay, as previously described [45].

#### 4.5.3. RNA Extraction and RT-PCR

As previously described [46], the total mRNA was isolated and prepared from the control and treated NCTC 2544 cells using the 1 mL Qiazol Reagent (Qiagen, Milan, Italy), 0.2 mL chloroform, and 0.5 mL isopropanol. An RNA pellet was washed with 75% ethanol, air-dried, and re-suspended in RNAse-free water. Reverse transcription was carried using the QuantiTect Reverse Transcription Kit (Qiagen), according to the manufacturer’s protocol. Synthesis of cDNA was performed using 40-cycle PCR in a Rotor-gene Q real-time analyzer (Corbett, Qiagen). The amplification of ICAM-1, iNOS, COX-2, NF-κB, and GAPDH was performed using specific primers listed in Table 4. Each PCR reaction contained one Rotor-Gene SYBR Green PCR Master Mix, template cDNA (≤100 ng/reaction), primers (1 μM), and RNase-free water, with a final reaction volume of 25 μL. RT-PCR was carried out according to the following program: initial activation step at 95 °C for 10 min, denaturation at 95 °C for 10 s, annealing at 60 °C for 30 s, extension at 72 °C for 30 s (40 cycles), and final extension at 72 °C for 10 min. RT-PCR was followed by melting curve analysis to confirm PCR specificity. Each reaction was repeated three times, and the threshold cycle average was used for data analysis by Rotor-gene Q software. The identification was carried out using electrophoresis in a 2% agarose gel in 0.045 M Tris–borate/1 mM EDTA (TBE) buffer. The target gene expression was normalized to GAPDH using the 2^−ΔΔCt^ method.

#### 4.5.4. Western Blot

The ICAM-1 and COX-2 protein expression levels were determined by Western blot analysis according to standard procedures [47]. The NCTC 2544 cells were stimulated with IFN-γ/H and treated with 50 µg/mL of *M. styphelioides* polyphenol extract for 72 h at 37 °C. Western blot was performed following the method reported [24]. Briefly, equal protein amounts were resolved by 4–12% Novex Bis–Tris gel electrophoresis (NuPAGE, InVitrogen, Milan, Italy), and transferred into nitrocellulose membranes (InVitrogen) in a wet system. The membrane was blocked in Tris-buffered saline containing 0.01% Tween-20 (TBST) and 5% non-fat dry milk for 1 h at room temperature. The membrane was incubated overnight with specific primary antibodies at 4 °C. Mouse monoclonal anti-ICAM-1 (1:200) (1H4: sc-51632; Santa Cruz Biothechnology), anti-COX-2 (1:300) (35-8200; Thermo Fisher Scientific), and anti-α-tubulin antibodies (Sigma-Aldrich) were used. Blots were later washed three times with PBS, followed by incubation in a HRP-conjugated secondary antibody for 1 h at room temperature. Specific proteins bands were detected using enhanced chemiluminescent solution (Pierce, Fisher Scientific) and visualized by a Uvitec Alliance LD9 gel imaging system (Uvitec, Cambridge, UK). Bands were measured densitometrically, and their relative density was calculated based on the density of α-tubulin bands in each sample. Values were expressed as arbitrary densitometric units (A.D.U.) corresponding to signal intensity.

### 4.6. Statistical Analysis

The experiments were repeated independently at least three times in triplicate, and the mean ± SEM for each value was calculated. One-way statistical analyses of the results (Student’s *t*-test for paired and analysis of variance (ANOVA) for unpaired data) were used. All statistical analyses were performed using the statistical software package SYSTAT, version 11 (Systat Inc., Evanston, IL, USA). A value of *p* < 0.05 was considered statistically significant.

## Figures and Tables

**Table 1:** Total phenols, flavonoids, and tannins content in *M. styphelioides* leaves.

M. styphelioides Extracts	Total Phenolic mg GAE/g Dry Extract	Total Flavonoid mg QE/g Dry Extract	Total Tannins mg Eq Catéchine/g Dry Extract
E. MeOH	142.7 ± 3.15	31.54 ± 1.99	15.2 ± 1.9
E. EtOAc	97.39 ± 7.69	26.8 ± 2.4	19.9 ± 2.9
E. Et_2_O	22.95 ± 0.4	7.83 ± 1.11	4.1 ± 1.3
E. Hex	3.27 ± 2.1	nd	nd

nd: not determined.

**Table 2:** Phenolic composition of M. styphelioides methanolic extract by LC/MS-MS.

C.A.S. Number	RT (min)	Mass (amu) [M − H^−^]	Fragments (*m*/*z*)	Compounds	Phenolic Family	Concentration (µg/kg DW)
327-97-9	0.59	353.8	191.20	Chlorgenic Acid	Phenolic acids	36 ± 7
149-91-7	1.28	169.01	125.00	Gallic Acid	Phenolic acids	1116 ± 127
1135-24-6	3.04	193.05	143.00	Ferulic Acid	Phenolic acids	86 ± 15
331-39-5	3.24	179.03	135.02	Caffeic Acid	Phenolic acids	92 ± 17
530-57-4	3.51	197.04	121.00	Syringic Acid	Phenolic acids	292 ± 35
207671-50-9	3.56	610.01	300.30	Rutin	Flavonoids	259 ± 31
520-26-3	3.59	609.20	301.00	Hesperidina	Flavonoids	177 ± 27
121-34-6	3.73	167.04	108.00	Vanillic Acid	Phenolic acids	359 ± 36
491-70-3	4.36	285.04	133.00	Luteolin	Flavonoids	56 ± 10
476-66-4	4.40	302.20	131.98	Ellagic Acid	Phenolic acids	522 ± 47
529-44-2	4.46	317.04	151.00	Myricetin	Flavonoids	160 ± 24
117-39-5	5.06	447.09	151.00	Quercetin	Flavonoids	4440 ± 355
67604-48-2	5.55	272.06	119.00	Naringenin	Flavonoids	23 ± 5
520-36-5	5.61	271.08	117.00	Apigenin	Flavonoids	336 ± 38
520-18-3	6.49	285.04	108.00	Kaempferol	Flavonoids	271 ± 35

**Table 3:** 2,2-diphenyl-1-picrylhydrazyl (DPPH) and 2,2′-azinobis-(3-ethylbenzothiazoline-6-sulphonate) (ABTS) scavenging activities, as well as the ferric reducing antioxidant power (FRAP) of *M. styphelioide*s leaf extracts.

*M. styphelioides* Extracts	DPPH IC_50_ μg/mL	ABTS IC_50_ μg/mL	FRAP mM FeSO_4_/g DE
E. MeOH	22.13 ± 2.17	21.39 ± 0.62	3.66 ± 0.014
E. EtOAc	119.15 ± 1.669	75.84 ± 1.22	0.85 ± 0.002
E. Et_2_O	73.24 ± 2.811	52.22 ± 1.40	nd
E. Hexane	229.9 ± 5.8	201.35 ± 9.4	nd
Trolox IC_50_	13.69 ± 0.04	64.37 ± 1.28	
BHT IC_50_	19.33 ± 0.32		

FRAP FeSO_4_·7H_2_O equivalent mM per gram of dry extract (mM/g DE). nd: not determined.

**Table 4:** Primers used in RT-PCR analysis.

Primers	Forward (5′→3′)	Reverse (5′→3′)
ICAM-1	GGCCGGCCAGCTTATACAC	TAGACACTTGAGCTCGGGCA
iNOS	GTTCTCAAGGCACAGGTCTC	GCAGGTCACTTATGTCACTTATC
NF-κB	ATGGCTTCTATGAGGCTGAG	GTTGTTGTTGGTCTGGATGC
COX-2	ATCATTCACCAGGCAAATTGC	GGCTTCAGCATAAAGCGTTTG
GAPDH	TCAACAGCGACACCCAC	GGGTCTCTCTCTTCCTCTTGTG

## Abbreviations

(H)	Histamine
(IFN-γ)	interferon gamma
(NF-κB)	nuclear factor kappa B
(ICAM-1)	inter-cellular adhesion molecule-1
(iNOS)	nitric oxide synthase
(COX-2)	cyclooxygenase-2
(DPPH)	2,2-diphenyl-1-picrylhydrazyl
(ABTS)	2,2′-azinobis-(3-ethylbenzothiazoline-6-sulphonate)
(E. hex)	hexane extract
(E. Et2O)	diethyl ether extract
(E. EtOAc)	ethyl acetate extract
(E. MeOH)	methanol extract
(M. MeOH)	*Melaleuca styphelioides* methanolic extract
